# Special Issue “Studies on Lactic Acid Bacteria and Their Products in Health and Diseases: 2nd Edition”

**DOI:** 10.3390/ijms27031514

**Published:** 2026-02-03

**Authors:** Ilavenil Soundharrajan, Balasubramani Ravindran, Ki Choon Choi

**Affiliations:** 1 Grassland and Forage Systems, National Institute of Animal Science, Rural Development Administration (RDA), Cheonan 31000, Republic of Korea; choiwh@korea.kr; 2 Department of Civil & Energy System Engineering, Kyonggi University, Suwon 16227, Republic of Korea; 3 Department of Biotechnology, Faculty of Engineering, Karpagam Academy of Higher Education, Coimbatore 641021, India

Lactic acid bacteria (LAB) provide essential benefits for both animal and human health due to their probiotic potential and their role in maintaining gut microbiome homeostasis. In humans, LAB, particularly *Lactobacillus* species and related genera, enhance digestive functions, modulate the immune system and support intestinal barrier integrity. In addition, LAB can suppress pathogenic microbial growth and increase the production of valuable secondary metabolites such short chain fatty acids while also contributing to vitamin synthesis and mineral metabolism. Supplementation of LAB as a probiotic significantly improves human health and reduces metabolic diseases and disorders such as obesity, sarcopenia, diabetes mellitus, liver cirrhosis and cardiovascular problems [1,2,3,4]. In animals, LAB are widely used as probiotics in livestock farming due to their ability to improve gut microflora balance, enhance nutrient absorption and digestion, strengthen mucosal integrity and help to prevent or control infections caused by pathogenic microbes through the production of antimicrobial substances [5,6]. Globally, LAB are also used as biological additives for the production of silage from grass and legume plants [7]. The addition of LAB during ensiling improves fermentation quality by increasing lactic acid content and reducing butyric acid level, both of which are most essential for high-quality silage production [8,9]. Also, LAB-mediated silage can improve animal performance, reduce the incidence of diarrheal diseases and improve overall health [10]. In recent years, lactic acid bacteria have attracted considerable interest due to their multifaceted roles, leading to extensive research on their mechanisms of action, therapeutic potential and safety [11]. This Special Issue highlights the growing global interest in LAB and underscores new opportunities and challenges in evaluating applications in health and livestock production.

## 1. About the Current Special Issue Collection

This Special Issue includes eight articles that illustrate a profound shift in understanding probiotics not merely as dietary supplements but also powerful biological agents capable of reshaping immune function, metabolic activity and agricultural ecosystems. These articles fall into three main categories: probiotics in toxicity reduction and immune modulation, their therapeutic potential for metabolic, viral, and gastrointestinal diseases, and microbiome-based interventions in agriculture and food systems.

### 1.1. Probiotics in Toxicity Reduction and Immune Modulation

A significant number of studies in this Special Issue demonstrate that probiotic exhibits protective effects against chemical, inflammatory and physiological stressors. Supplementation with *Levilactobacillus brevis* 47f in zebrafish significantly reduced toxic effects, resulting in increased survival rates in zebrafish challenged with imidacloprid and a significant reduction in histological lesions in the intestine and kidneys. The strain supplement also exhibited multidirectional effects on various anti-inflammatory cytokines. This work supports *L. brevis* 47f as a protective agent against agricultural contaminants and demonstrates that targeted probiotics can alleviate pesticide-induced toxicity in aquatic animals (Contribution 1). Another study revealed that *Lactiplantibacillus plantarum* HY7718 treatment attenuated dextran sulfate sodium (DSS)-induced ulcerative colitis in a mouse model. HY7718 supplementation significantly improved the disease activity index score and histological index and also prevented DSS-induced weight loss. HY7718 treatment upregulated the expression of intestinal tight junction-related genes and downregulated pro-inflammatory cytokines and genes involved in the TLR/MyD88/NF-kB signaling pathway, recommending that HY7718 may be considered a functional supplement for improving inflammatory bowel disease (Contribution 2). Similarly, *L. plantarum* P101 supplement for 3 weeks resulted in significant improvements in liver injury, oxidative stress and inflammation induced by cyclophosphamide. In addition, P101 supplementation enhanced the antioxidant defense system by activating the Nrf2/ARE signaling cascade and protected normal liver functional, suggesting that P101 is a promising strain to alleviate Cyclophosphamide-induced hepatotoxicity (Contribution 3).

### 1.2. Therapeutic Potential for Metabolic, Viral, and Gastrointestinal Diseases

Three articles in this section demonstrate the therapeutic potential of probiotics for viral infections, metabolic dysfunction and gastrointestinal injury. The effects of *Lactiplantibacillus paraplantarum* BGCG11 on pancreatic and intestinal function were evaluated in streptozotocin-induced diabetic rats. Biochemical markers of diabetes significantly improved after probiotic treatment. In addition, BGCG11 supplementation restored islet morphology, prevented tissue damage and enhanced insulin production. Collagen deposition and inflammation in the duodenum were reduced. BGCG11 treatment also improved mucosal integrity, and gut microbiota shifted toward more beneficial taxa after administration. This study underscores the probiotic’s potential in managing diabetes-related tissue dysfunction (Contribution 4). Another study revealed that *L. plantarum* expressing a PRRSV single-chain antibody inhibited PRRSV replication and beneficially modulated the gut microbiota in piglets. Transcriptomic analysis indicated enhanced antiviral pathways in treated animals, highlighting the recombinant probiotic’s ability to reduce viral replication and boost immune responses (Contribution 5). A review article further emphasizes the pivotal role of gut dysbiosis in liver inflammation and highlights microbiota-targeted therapies as emerging treatment strategies, suggesting that probiotics can modulate metabolic pathways, enhance antiviral immunity, and improve gastrointestinal function (Contribution 6).

### 1.3. Microbiome-Based Interventions in Agriculture and Food Systems

Beyond laboratory and clinical research, these articles underscore the critical role of the microbiota in modern agricultural and food production practices. Studies such as ‘Dietary fermentation with *Lactobacillus* spp. and *Bacillus* spp. modulates rumen transcriptomic and microbiota Profiles in *Bos taurus’* (Contribution 7) and ‘Genomic and phenotypic characterization of mastitis-causing Staphylococci and probiotic lactic acid bacteria isolated from raw Sheep’s milk’ (Contribution 8) demonstrate how microbial communities influence livestock health, milk safety and overall animal performance. Jung et al. reproted that diet supplementation fermented with *Lactobacillus* and *Bacillus* altered gene expressions in major metabolic organs including the adipose, liver and muscle tissue in Hanwoo steers. Most of the differentially expressed genes in major metabolic organs were associated with osteoclast differentiation and immune functions. Bacterial community analysis revealed that the fermented diet significantly increased *bacillota* while reducing *bacteroidota* abundances. Fungal diversity in the rumen also shifted notably in response to fermented feed. These microbial changes correlated with enhanced nutrient utilization. The authors highlight improved cattle performance driven by fermentation-based dietary modifications, supporting fermented feed as a promising strategy for enhancing productivity. Apostolakos and colleagues characterzed mastitis-causing staphylococci and probiotic lactic acid bacteria from raw sheep’s milk. The probiotic LAB strains displayed favorable phenotypic traits and wholegenome sequencing revealed subtantial strain-level diversity among isolates. This work enhances understanding of microbial ecology in sheep milk and emphasizes the dual challenge of pathogenic bacteria and beneficial microbial, supporting the development of preventive strategies against mastitis causing staphylococci in dairy production. Collectvely, these finding point out a future in which microbiome management become as integral to sustainable farming as nutrition and genetics.

## 2. Significance of This Special Issue

Collectively, these articles address several important research gaps across probiotic science, host–microbe interactions and disease mitigation. Prior to these studies, limited evidence existed on how specific lactic acid bacteria counteract environmentally relevant toxicants such as pesticides or chemotherapy agents through integrated assessments of immune balance, organ histology and microbial restructuring. Similarly, the therapeutic potential of probiotics in complex systemic diseases such as alcoholic hepatitis, diabetes related tissue dysfunction and colitis had not been fully explored with multi-omics or mechanistic depth, leaving gaps in understanding how microbial modulation translate into organ-level protection. In livestock and aquaculture, there was a noticeable lack of research on engineered probiotics as antiviral delivery systems and how a fermented diet modulates both microbial communities and host gene expression to improve productivity. Additionally, the microbial ecology of raw sheep’s milk, especially the interplay between pathogenic staphylococci and beneficial lactic acid bacteria, remained insufficiently characterized at the genomic level. By integrating immunology, microbiome profiling, transcriptomics, metabolomics, and functional assays, these studies fill gaps in mechanistic clarity, strain-specific efficacy, cross-organ communication, and ecological understanding. Together, this Special Issue advances the field by providing deeper insight into how probiotics can serve as sustainable, targeted and biologically refined interventions across environmental, clinical and agricultural research.

## 3. Future Research

It is important that future research deepens our understanding of how LAB act as probiotics to modulate host immunity, metabolism, and barrier function across multiple disease models. Multi-omics integration will be essential to link microbial shifts with molecular, cellular, and physiological outcomes. Research needs to be focused on comparative studies across aquatic, rodent, livestock, and human species to clarify both conserved and host-specific mechanisms of probiotic action. Long-term and dose–response experiments should also be conducted to determine durability, safety, and the optimum delivery strategies. To maximize resilience against toxins, infections, metabolic dysfunction, and inflammatory diseases, future research should investigate the synergistic interactions between probiotics, prebiotics, fermented diets, and conventional therapies. It is also essential to explore strain- and host-specific probiotic compositions supported by well-defined mechanistic evidence. Translational studies, such as controlled clinical trials and field applications in agriculture and aquaculture, are required to validate laboratory findings and assess real-world effectiveness. To advance probiotics as sustainable, biologically driven solutions for health and disease management, future research work should prioritize designing precise, mechanism-based probiotic interventions tailored to specific environmental stressors, pathogens, and host conditions.

## 4. Conclusions

These diverse articles demonstrate that probiotics exert a wide range of benefits that extend far beyond gut colonization, making them effective biological tools for disease treatment, health restoration, and environmental resilience. Evidence from aquatic, livestock and mammalian models shows that targeted probiotic strains can counteract chemical toxicants and infections agents, metabolic dysfunction and inflammatory damage by simultaneously modulating immune pathways, enhancing epithelial integrity, reducing oxidative stress and restructuring the gut microbiome. Strains such as *L. brevis* 47f demonstrate the ability to protect against environmental pollutants like imidacloprid by reinforcing organ histology and normalizing inflammatory cytokines, while *L. plantarum* showcases how probiotics can be engineered as living delivery platforms for antiviral therapeutics. Probiotics such as *L. plantarum* BGCG11 and HY7718 effectively restore barrier function, rebalance cytokine networks, improve antioxidant responses and reshape microbial communities towards beneficial profiles, highlighting their promise in managing diabetes, hepatotoxicity and colitis. Studies in ruminants further illustrate how a fermented diet with *Lactobacillus* and *Bacillus* can reshape rumen microbiota and host gene expression to improve nutrient utilization and overall productivity. Additionally, investigations into sheep’s milk microbiota underscore the interplay between pathogenic and beneficial bacteria, emphasizing the importance of probiotic LAB in dairy safety and fermentation technologies. Collectively, these findings reveal that probiotics influence host biology at molecular, cellular, and ecological levels, functioning as sustainable, adaptable, and mechanistically diverse interventions. Therefore, we invite researchers to explore the findings presented in this Special Issue, which collectively underscore the remarkable potential of LAB as natural, safe, and effective agents in supporting human and animal health.

## Data Availability

Not applicable.
